# Could You Give Me the Blue Brick? LEGO^®^-Based Therapy as a Social Development Program for Children with Autism Spectrum Disorder: A Systematic Review

**DOI:** 10.3390/brainsci11060702

**Published:** 2021-05-26

**Authors:** Antonio Narzisi, Gianluca Sesso, Stefano Berloffa, Pamela Fantozzi, Rosy Muccio, Elena Valente, Valentina Viglione, Arianna Villafranca, Annarita Milone, Gabriele Masi

**Affiliations:** Department of Child Psychiatry and Psychopharmacology, IRCCS Stella Maris Foundation, 56018 Pisa, Italy; gianluca.sesso@fsm.unipi.it (G.S.); stefano.berloffa@fsm.unipi.it (S.B.); pamela.fantozzi@fsm.unipi.it (P.F.); rosymuccio@yahoo.com (R.M.); elena.valente@fsm.unipi.it (E.V.); valentina.viglione@fsm.unipi.it (V.V.); arianna.villafranca@fsm.unipi.it (A.V.); annarita.milone@fsm.unipi.it (A.M.); gabriele.masi@fsm.unipi.it (G.M.)

**Keywords:** autism, LEGO^®^-based therapy, children, social skills, intervention, systematic review

## Abstract

LEGO^®^-based therapy is a social skills development program aimed at children with autism spectrum disorder (ASD). A systematic review of the literature was conducted using PRISMA guidelines. PubMed, Scopus and Web of Science bibliographic databases were searched from their date of inception to August 2020. The review included 19 studies. Studies were classified according to experimental designs (e.g., Randomized Control Trial, Non-Randomized Studies of Interventions and case report and series) and a narrative synthesis of each was provided, along with a critical discussion of the strengths and weaknesses of the available literature on the topic. Although LEGO^®^-based therapy appears a promising treatment for social interaction in ASD, the findings of LEGO^®^-based therapy studies should be interpreted and generalized with caution, due to the low quality of the studies and the small sample sizes.

## 1. Introduction

Autism spectrum disorder (ASD) is a multifactorial and heterogenous disorder characterized by an umbrella of specific issues in the areas of social communication, restricted interests and repetitive behaviors [1,2,3]. In the last two decades, the diagnosis of ASD has significantly increased worldwide and recent epidemiological data estimated it to be higher than 1/100 [1,4,5,6].

In the last years, the idea of ASD, both in terms of diagnosis and treatment, has been reconsidered, and this reconceptualization included not only the difficulties, but also the exceptional abilities that can distinguish people living with ASD [7,8]. Among the core issues of ASD, social skills are mostly prominent [9]. Indeed, children with ASD exhibit difficulties in establishing friendships and playing cooperative games [10], and they are consequently poorly included in social life [11] and are more vulnerable to demoralization, depression [12], anxiety [13] and low self-esteem [14]. Given that social participation is a key predictor of quality of life and overall functioning, it is crucial to enhance the social functioning of these children [15]. Many interventions aiming to improve social abilities have been studied, but few of them are evidence-based [16] and/or have reliable and valid methods for measuring social skills [17].

Although guidelines for social skills interventions have been published over the years, few of them showed evidence of efficacy [18,19,20], and the focus was often on modifying deficiencies rather than building the points of strength [21,22,23]. Among the few recognized social skills development interventions [24,25], such as Social Stories [26] and PEERS^®^ [27], LEGO^®^-based therapy [23] is used with the aim to improve social interactions and collaborative play in multiple settings [28,29].

LEGO^®^-based therapy is a social development intervention for young people with ASD or related social communication difficulties, originally developed by LeGoff in 2004 [23]. LEGO^®^-based therapy can be used individually or in groups, and natural opportunities for developing social skills are facilitated by the therapist.

As reported by Gallo-Lopez and Rubin [30], LEGO^®^-based therapy can be allocated among the so-called play-based interventions. These types of interventions for improving social communication skills in ASD can be divided into those that focus on improving social play skills in their own right, and those, such as LEGO^®^-based intervention, that use playful methods to target other social outcomes.

The research on these play-based interventions, however, varies greatly, including differences in underpinning theoretical approaches, modes of delivery and conceptualizations of play, as well as in the designs used to assess their effectiveness. Some interventions take a more instrumental approach, while others, such as LEGO^®^-based intervention, emphasize following a child’s voluntary play interests.

As Gibson and colleagues [31] highlighted in a recent literature review, the types of interventions that LEGO^®^-based therapy shares with other play-based interventions are: (1) *feedback-based* (practitioners provide tailored feedback to target a child and/or their interaction partner during or after play; the play partner may be an adult, a peer or a teacher); (2) *social skills group* (delivered to a group of children; they often support the development of holistic or specific social and communication skills); (3) *activity schedule* (interventions which use steps related to a target activity to support the child’s successful or appropriate involvement) (4) *collaboration-based* (collaborative working with others for a task within an intervention to be successful).

The most widely used method of LEGO^®^-based therapy involves at least three participants, each taking a turn playing one of three roles: “supplier”, “builder” or “engineer” [31]. The supplier’s role is to locate and retrieve the blocks as instructed by the engineer, who is responsible for interpreting the instructions and determining which pieces are needed for each step of the assembly. The builder is responsible for assembling the blocks according to the instructions given by the engineer [31]. LEGO^®^-based therapy is not the expression of a particular theoretical frame of reference, but rather a form of mixed naturalistic developmental behavioral intervention that has been conceived based on the natural interest of the children, particularly those with ASD, for buildings [23,32,33]. Around this interest, which especially in children with ASD could represent an alternative to social relationships, the authors of LEGO^®^-based therapy have developed a program for social skills development [31]. The core of the therapeutic intervention is a collaborative process, with an intrinsic interdependence, which creates an environment in which attention to the group, sharing of objectives, turn-taking, group results, interpersonal relationships and mutual respect, positive moods and appreciation are necessary [31,34,35].

The first documented experience of LEGO^®^-based therapy was described by two studies published in 1995. Altman and Esber [36] first used LEGO^®^-based therapy on nine adolescents (11–16 years old) with severe disruptive behavior disorders: in this seminal work, the authors underlined the effects of this intervention on focus, attention and collaborative skills. Kohler [37] adopted manipulative play activities, including LEGO bricks, with three preschoolers with ASD and six of their typical classmates, in order to study the group-oriented contingency to increase social interactions between children with ASD and their peers. This study showed that the interdependent group contingency improved the socio-interactive exchanges between children with ASD and peers.

Since then, a discrete amount of LEGO^®^-based therapy studies have been conducted. The main aim of our review is to summarize the available data of LEGO^®^-based therapy studies to understand the effect on social skills in children with ASD.

## 2. Methods

### 2.1. Search Strategy and Review Process

A systematic review of the literature was conducted using PRISMA (Preferred Reporting Items for Systematic Reviews and Meta-Analyses) guidelines [38] and the corresponding checklist is available in Supplementary Table S1. PubMed, Scopus and Web of Science bibliographic databases were searched from their date of inception to August 2020. Reference lists of all included studies were also searched for further relevant citations. The authors discussed and reviewed the results of an initial scoping search and finally used the following search strategy: (LEGO therapy) AND (autism OR autistic AND disorder); the full search strategy is available in Supplementary Table S2. The search included reviews and original studies and, if a previous review was found, the reference list was searched to identify and retrieve the primary studies. Abstracts were retrieved using our search strategy and duplicates were then removed.

Inclusion criteria for our systematic review were as follows: (1) participants: children and/or adolescents with a diagnosis of ASD; (2) intervention: LEGO^®^-based therapy; (3) controls: no a priori limitations were applied; (4) outcomes: studies providing quantitative outcomes based on clinical measures of social skills; and (5) study design: any clinical interventions (e.g., Random Controlled Trials, Non-Randomized Studies of Interventions, case series and studies, etc.). Exclusion criteria were as follows: (1) no quantitative outcome measures were provided (i.e., studies providing qualitative results based on clinical observations); (2) articles published in a language other than English; (3) studies providing protocol designs rather than clinical interventions; and (4) reviews. Three researchers (A.N., R.M. and G.S.) screened all titles and abstracts to identify relevant articles for full-text retrieval. Any disagreements were resolved by consensus.

When datasets of any study were not fully available, authors were contacted to attain the required data and include all possible studies. According to the study design, the studies were subdivided into RCT, when participants were randomly assigned into an experimental group or a control group; non-randomized longitudinal studies, when subjects were not randomly assigned (quasi-experiment); waiting list control and within-subjects baseline design, when a group of participants did not receive the experimental treatment and when participants served as their own control by providing baseline scores across different conditions (quasi-experiment); and case reports and series, when a control group was not included (non-experimental).

### 2.2. Identification and Selection of Studies

**Figure 1:**
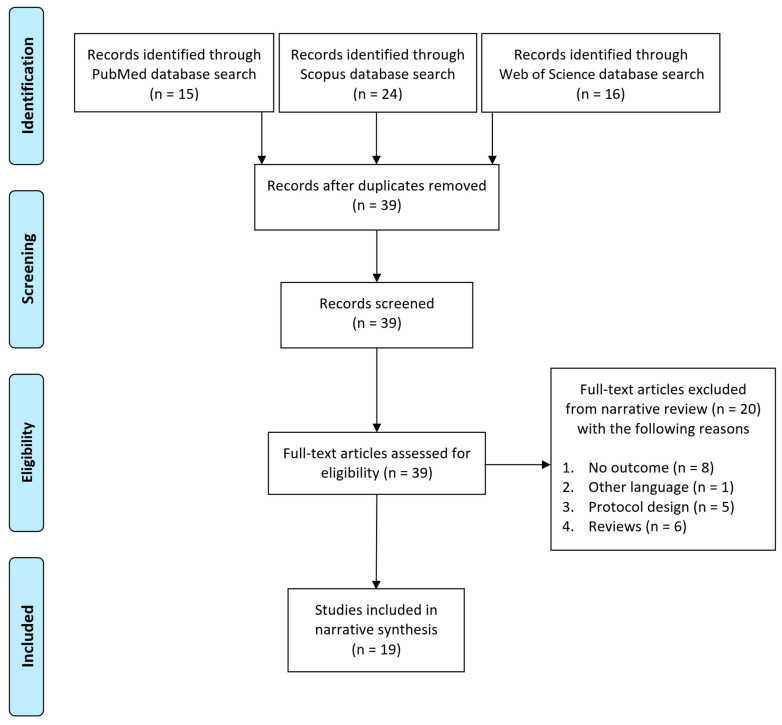
Process of identification and selection of papers.

Figure 1 shows the process of identification and selection of papers, starting from 55 abstracts that were retrieved using our search strategy. First, 16 abstracts were removed as duplicates; thus, 39 were carefully screened. Twenty records were excluded based on abstract or title. The remaining 19 full-text studies were included in the narrative review and subdivided according to the type of study and level of evidence (Table 1).

### 2.3. Quality Assessment

The quality of all the studies was ascertained using the JHNEBP (John Hopkins Nursing Evidence Based Practice). It permits us to evaluate: (a) the strength of evidence through five levels ranging from RCT (Level I) to case report and case series (Level V); and (b) the quality of evidence ranging from high to low [39].

Moreover, the ROBIN-I (Risk of Bias In Non-Randomized Studies—Of Interventions) [40] and RoB-2 (Risk Of Bias for Randomized Studies) [41] scales of the Cochrane risk of bias tools for non-randomized studies (NRSI) and RCT were used, respectively. For ROBIN-I, seven risk of bias items were individually assessed by answering each question that determines low, moderate, serious, critical or no information of bias. Low indicates lower risk of bias of the studies and critical indicates higher risk of bias of the studies [42]. For what concerns the RoB-2, six risks of bias items were individually assessed in the RCT by answering each question that determines the high (H), unclear (U) or low (L) risk of bias. High risk of bias indicates low quality of studies. Low risk of bias indicates high quality of studies. Unclear risk of bias indicates that the limited information restricted correct judgment to identify low or high quality of the studies [43,44].

## 3. Results

### 3.1. Narrative Review of Included Studies

#### 3.1.1. LEGO^®^-Based Therapy: RCT

In the study conducted by Owen and colleagues [29], LEGO^®^-based therapy has been compared with another approach, the SULP (Social Use of Language Program) [45]. Both interventions were administered to children aged 6–11 years old with ASD. Participants were randomly assigned to LEGO (n = 16) or SULP (n = 15). The results show that the LEGO^®^-based therapy group improved more than the comparison group on the social interaction sub-scale of the Gilliam Autism Rating Scale (GARS). No significant improvement was detected in both SULP and LEGO groups in terms of communication and socialization skills

#### 3.1.2. LEGO^®^-Based Therapy: Non-Randomized Studies of Interventions (NRSI)

In 2006, LeGoff and Sherman [28] conducted a 3-year retrospective study of long-term outcomes on 60 children with ASD participating in LEGO^®^-based therapy. The authors compared pre- and post-treatment measures (the socialization domain of Vineland Adaptive Behavior Scales (VABS) and social interaction subscale of GARS). A control group of 57 children with ASD, matched for age and sex, who received comparable non-LEGO^®^-based therapy from other providers was recruited. Both groups showed significant gains in the two outcome measures (GARS and VABS); however, LEGO^®^ participants improved significantly more than the comparison subjects.

In 2015, the study of Huskens [46] and colleagues investigated robot-mediated LEGO^®^-based therapy with three children with ASD (aged 5–10 years old) and their three siblings (aged 7–11 years old). The intervention consisted of five 30-min weekly sessions lead by the robot instead of the trainer. The trainer was present during all sessions to control the robot at a laptop and to assist the robot when needed. No statistically significant changes were found in the collaborative behaviors of the children with ASD. However, this study provided several practical implications and directions for future research.

In 2016, Peckett, MacCallum and Knibbs [47] explored the use of LEGO^®^-based therapy in a home-setting by five mothers of children with ASD (aged 5–16 years) for 6 weeks. A total of 10 children in 5 sibling pairs participated (six with ASD and four without ASD). Using interpretative phenomenological analysis, improvements were reported in family relationships. As reported by the authors [47], some ambivalence about the impact of the intervention in the wider context emerged.

In 2018, Hu and colleagues [48] examined the effects of LEGO^®^-based therapy on children with ASD in an inclusive preschool. Three male preschool children with high functioning ASD, aged 4–6 years old, and 13 typically developing children (three girls and ten males, aged 4–6) participated as peers in this research. The intervention consisted of LEGO construction activities incorporated with peer-mediated strategies for one child with ASD and two typically developing peers. As described by the authors, the results indicated the adequacy of the social validity of the intervention and all three children with ASD increased their social reciprocity.

In 2020, Levy and Dunsmuir [49] explored the impact of school-based LEGO^®^-based therapy on six male adolescents (aged 11–14 years old) with ASD in mainstream schools. In addition to the six participants with ASD, 12 typically developing peers were recruited via volunteer sampling. Two typically developing peers and one student with ASD formed each LEGO Club group, with data collected only for the student with ASD. School staff were trained in the social skills. The LEGO Club intervention was adapted from classical LEGO^®^-based therapy interventions as implemented by LeGoff et al. 2004 [23]. LEGO Club sessions occurred twice a week for a total of 12 intervention sessions. Social behaviors were coded using a social behavior coding schedule. The coding scheme allowed the observation of the quality (positive or negative) and nature (initiating or responding) of social behaviors. The duration of social interactions was also recorded and calculated as a percentage of time engaged with peers. The results showed a significant effect of LEGO therapy on social engagement and frequency of social initiations, responses and positive social behaviors for five out of six participants. Parents and teachers reported some evidence of skills generalization at home and also in several school settings. LEGO-based therapy fidelity was maintained by the trained school staff.

#### 3.1.3. LEGO^®^-Based Therapy: Waiting List Control and Within-Subjects Baseline Design

In 2004, LeGoff [23] implemented the use of LEGO^®^-based therapy as a specific intervention for children with ASD. Waiting list control design, with repeated measures, was used to assess the efficacy of LEGO^®^-based therapy on individual and group LEGO play. The intervention, addressed to 47 children with ASD (34 males and 13 females, aged 6–16 years; mean age = 10.6 years, standard deviation (SD) = 2.8), combined aspects of behavioral therapy, peer modeling and naturalistic communication strategies. Children who participated in the study were involved in an individual therapy session (lasting 60 min) and in a LEGO^®^ therapy session (90 min). All 47 children in the study had been on a waiting list for treatment for at least three months, and 21 of these were on a waiting list for at least 6 months. The design utilized a waiting list control group, with repeated measures, beginning with an intake assessment, prior to being placed on the waiting list. Consequently, all 47 subjects were able to serve as their own control group for a 3-month treatment trial, and 21 of them were able to serve as a control group for a 6-month treatment period [23]. The results revealed a significant improvement in (1) social motivation and interaction with peers as revealed by unstructured observations; and (2) shared activities as revealed by the GARS.

In 2012, Andras [50] studied the improvement resulting from ten weekly sessions of LEGO^®^-based therapy in primary-school-aged children (6–11 years old; seven males; one female) with ASD. The study adopted a waiting list control design. During the ten-week intervention period, a 45 min session was delivered each week by the school staff. The findings showed that the social interaction between children was improved after the LEGO^®^ therapy and this effect was maintained even after the end of the therapy.

In 2013, Brett [51] explored the improvements resulting from LEGO^®^-based therapy in a school setting in children with Asperger disorder using a within-subjects baseline design. A total of 14 participants (13 males and 1 female aged 9.07 ± 1.3) were involved. The LEGO^®^-based therapy sessions occurred 45 min once per week in school for nine weeks. Significant improvements in socio-interactive competencies (evaluated via VABS) were seen after participation in LEGO^®^-based therapy. There were not found statistical differences in the communication domain of VABS.

#### 3.1.4. LEGO^®^-Based Therapy: Case Reports and Series

In 2010, Pang [52] reported the use of LEGO^®^-based therapy in a child (3 years old) with ASD. A self-developed observation checklist was used to monitor the child’s social emotional development, fine motor skill and language acquisitions, as well as challenging behaviors. After three LEGO^®^-based therapy sessions, the child increased his social interactions (i.e., increased interest in playing with peers, shared blocks and toys with peers and expressed more social motivation), developed a longer attention span, started to answer questions when asked and improved his verbal communication abilities (i.e., an expressive vocabulary grown from 30 to 60 words and simple sentences were produced).

In 2010, Wainer [53] described an exploratory study using LEGO and robotics involving seven children (aged 8–14 years) with ASD. In this class-setting study, children and their peers programmed LEGO robots under the guidance of an experimenter. The results showed improved collaborative behaviors among children. In addition, many children found their experience in the class helpful for other social interactions.

In 2012, Andrew [54] and colleagues used LEGO^®^-based therapy on a 8-year-old child with ASD. The child was involved in 75-min weekly individual sessions of LEGO^®^-based therapy. Significant improvements were obtained in the communication domain.

In 2014, Evans [55] explored the use of LEGO^®^-based therapy in 18 males (6–11 years old) with ASD. The intervention consisted of eight weekly group sessions guided by a therapist in a clinic setting. The results showed active participation of children in the LEGO^®^ sessions and parental satisfaction with the intervention.

In 2014, Tuonen [56] and colleagues investigated triadic interactions between children with ASD during a technology-enhanced LEGO building activity. In their pilot study, the authors applied a LEGO^®^-based therapy intervention on four children (two boys and two girls aged 8–13 years old) with ASD, using video data recorded in a natural technology-enhanced environment. Children communicated with augmentative and alternative communication (AAC) methods at school, and teachers reported that these children required extensive support. This pilot study was targeted to children’s behaviors at the LEGO building station, to support collaboration between children and adults based on previous research. At the LEGO building station, the children were presented with a model on a computer screen, which they were instructed to recreate using LEGO and DUPLO bricks. The children could choose to either (1) build the construction from a complete model (either figures or abstract models), (2) build the model step-by-step or (3) play a memory game in which the model was hidden during the building phase. The game was based on the LDraw™ open standard for LEGO CAD (Computer-Aided Drafting) programs. The main findings were obtained in terms of positive effects and joint attention. The results of this study suggested that an interesting environment or equipment might increase the emergence of triadic interactions among children with ASD.

In 2014 Boyne [57] studied the effects of LEGO^®^-based therapy on six children, aged 6 to 10, with social communication difficulties. Sessions were recorded and the videos were coded using an adapted version of Thunberg, Ahlsen and Sandberg’s Communication Coding Scheme, to explore the participant’s social confidence and independence, development and maintenance. Pre-treatment, post-treatment and delayed outcome measures, evaluated by means of the Social Competence Inventory (SCI) and the Belonging Scale, assessed the participants’ parents’ and teachers’ perceptions of skill generalization, and the participants’ self-reported sense of school belonging. The results showed that the children improved in at least one social communication skill, and this was maintained after the intervention in three participants. An increase in the perceptions of the participants’ social communication skills was reported within the school (five out of six) and home environment (three out of six). All participants rated a high level of sense of school belonging prior to the intervention, and change was variable among participants following the intervention.

In 2015, Barakova [58] and colleagues applied LEGO^®^-based therapy through a robot for children with ASD, with six male children aged 8–12 years old. The intervention was delivered by a therapist in a clinical setting and consisted of 30-min weekly sessions for one month. Significant findings in social initiative and decreased episodes of playing alone were shown.

In 2015, MacCormak et al. [59] studied 17 male children with neurodevelopmental disorders (including 12 with ASD) aged 7–12. LEGO^®^-based therapy was applied in a community-based program for four weekly one-hour sessions. The results showed increased play and socialization skills.

In 2015, Yalamanchili [60] assessed the social skills outcomes of LEGO^®^-based therapy in six preschoolers with ASD. The children were divided into two groups, one treatment group and one control group. The Autism Spectrum Rating Scales (ASRS) scores were reported for each participant at pre-, peri- and post-intervention (eight weeks after the program began). The results showed that the treatment group failed to make significant social skill gains more so than the participants in the control condition. However, they did make individual improvements in social skills as defined by the ASRS.

In 2016, Griffiths [61] studied the experience of teachers and parents with LEGO^®^-based therapy. Four LEGO^®^-based therapy groups were established with the aim of facilitating the social competence skills of 13 children (aged 7–12 years) with ASD. Six teachers and seven parents were recruited to participate in the research. School staff completed the GARS-2 to measure the children’s social-communication skills before and at the end of the intervention. GARS-2 scores did not demonstrate a significant effect of intervention over time. The findings demonstrated that teachers perceived that there had been domain-specific gains in social skills when engaging with LEGO materials, but noted a lack of generalization of skills from therapeutic to non-therapeutic contexts. Parents perceived an increased interest in LEGO materials as well as improved communication and initiation of interaction at home, suggesting that an element of skill generalization had been achieved.

### 3.2. Strength, Quality of Evidence and Risk of Bias Assessment

According to the JHNEBP-evaluated strength of evidence, only one study was of Level I [29], eight were of Level II [23,28,46,47,48,49,50,51], eight were of Level III [53,55,56,57,58,59,60,61] and two were of Level V [52,54]. According to the JHNEBP-evaluated quality of evidence, seventeen studies were of low quality of evidence [23,46,47,48,49,50,51,52,53,54,55,56,57,58,59,60,61] and only two were of good quality of evidence [28,29] (see Table S3 in the Supplementary Materials for detailed evaluation). Only one study, of Owens and colleagues [29], was an RCT and the risk of bias was Low using RoB-2 (see Table S4 in the Supplementary Materials for detailed evaluation). Five studies were NRSI and the risk of bias was evaluated using ROBIN-I. One study [28] showed a moderate risk of bias and four studies [46,47,48,49] showed a critical risk of bias (see Table S5 in the Supplementary Materials for detailed evaluation).

## 4. Discussion

The present study was aimed at systematically reviewing the available literature assessing the effectiveness of LEGO^®^-based therapy interventions on youth with ASD. The review included 19 studies, published over a 16-year period, each including from 3 to 60 participants aged from 3 to 16 years across five countries. Among the studies reviewed in our paper, twelve of them were conducted without a control group [50,51,52,53,54,55,56,57,58,59,60,61].

From the assessment of individual studies, a great variability emerged in the number of treatment sessions applied to subjects with ASD, ranging from 3 [50] to 31 [46]. Despite this, LEGO^®^-based therapy has shown some consistency, with encouraging results in terms of effectiveness. The main positive outcome, transversely described by the studies, was the empowerment of social skills in treated subjects [23,28,29,45,47,49,51,52,53,55,57,58,59]. Nonetheless, studies also reported a positive impact on autistic symptoms such as aloofness and rigidity [54], an enhancement in verbal communication abilities [29,46,51,56] and improved family relationships [51]. Moreover, as reported by Tuonen [55], the joint use of LEGO bricks with technological applications of various kinds [60,61] has been found of interest for people with ASD.

The flexibility of LEGO^®^-based therapy makes it possible to be easily applied at home [51], in clinical settings [23,29,45,46,53,54] and/or at school [47,49,52,55,56,57,58]. As clarified in the LEGO manual by Legoff [23], the intervention also provides the possibility of adopting different methodologies for implementing the sessions. Thus, LEGO^®^-based therapy can be considered as (1) an individual therapy for the implementation of developmental skills (i.e., for younger children, or for those with cognitive and visual-motor disorders); (2) a group therapy in collaboration with a partner; (3) a group therapy in collaboration with two companions; and (4) a social communication/individual therapy (i.e., to work on specific behavioral or communication issues).

According to reports by the American Academy of Pediatrics (AAP) and the National Research Council (NRC), LEGO^®^-based therapy can be considered social skill training because it (1) teaches children the skills they need to interact with others, including conversation and problem-solving skills; and (2) it provides structure, direction and organization for the child in addition to potential family participation; it can be peer-mediated; and it could improve friendship quality [62].

The findings of LEGO^®^-based therapy studies should be interpreted in light of several limitations. First, we found only one RCT and the overall quality of the included studies was low. Future RCT should be implemented to clarify whether LEGO^®^ -based therapy is more effective than standard care or other psychosocial therapies for ASD. Second, several different measures of social skills were used in the different studies, which partially limited the comparability of results. Third, the sample size of the studies was small. Fourth, there was an extreme variability in terms of clinical and socio-demographic characteristics [15,63]. Fifth, we decided to use a relatively narrow search strategy to identify only studies assessing the effects of LEGO^®^-based therapy on ASD; following this, some studies may have been missed if they used terms such as neurodevelopmental disorders or social communication problems; however, on the other hand, our decision allowed us to increase the specificity of the intervention target. Sixth, the systematic review was focused only on outcomes related to social interaction, but other outcomes that might benefit from LEGO^®^ therapy could be potentially evaluated (e.g., joint attention, fine motricity, etc.). Seventh, the protocol of our systematic review was not preregistered on an international database such as PROSPERO (Prospective Register of Systematic Reviews).

For these reasons, caution should be used in generalizing the findings. In agreement with Lindsay [15], future research trials should include greater sample sizes and more rigorous RCT designs, along with standardized measures. Interestingly, in 2019, Varley [64] and colleagues published a promising study protocol describing their forthcoming multicenter, pragmatic and RCT study, which will contribute to the achievement of more significant results to be generalized.

## 5. Conclusions

The usability of LEGO^®^-based therapy in different settings can be considered the strength of this approach, and the possibility of also involving typically developing peers and parents may represent a naturalistic learning and sharing opportunity for people with ASD [65]. However, more clinical studies need to be conducted to make LEGO^®^-based therapy a recommended evidence-based intervention.

## Figures and Tables

**Table 1:** Studies included in the systematic review.

Year	Author	Sample	Age	Setting	Time/Session	Tools	Results	Treatment Duration	SoE ^1^	QoE ^1^	RoB/ ROBINS-I ^2^
2004	LeGoff	47 (34 M; 13 F)	6–16	Clinical	1 h week/individual+ 1.5 h/week/group	Observation+ GARS	Improved on (1) social motivation; (2) interaction; and (3) shared activities	24 weeks	II	Low	NC
2006	LeGoff and Sherman	ASD-exp (49 M; 11 F) ASD-ctr (47 M; 10 F)	9–10	Clinical	1 h week/individual+ 1.5/h/week group	GARS-SI VABS-SD	Improved on social development	3 years	II	Good	Moderate ^††^
2008	Owens et al.	ASD-exp (16 M) ASD-ctr (15 M; 1 F)	6–11	Clinical	1 h/week	GARS-SI	Improved on socialization	18 weeks	I	Good	Low ^†^
2010	Pang	1 M	3	School	5 h/week	Self-developed observation checklists	Improved on social interactions and verbal communication ability	3 months	V	Low	NC
2010	Wainer et al.	7 M	8–14	School	1 h/week	Interviews, questionnaires and videoobservation	Improved on social interaction	11 weeks	III	Low	NC
2012	Andras	8 (7 M; 1 F)	6–11	School	45 m/week	Self-developed observation checklists	Improved on social interaction	10 weeks	II	Low	NC
2012	Andrews et al.	1 M	8	Clinical	75 m/week	Self-developed observation checklists	Improved on communication	24 weeks	V	Low	NC
2013	Brett	14 (13 M; 1 F)	9	School	45 m/week	VABS	Improved on socialization, play and interpersonal skills	9 weeks	II	Low	NC
2014	Evans et al.	8 M	6–11	Clinical	1 h/week	Questionnaire with Likert scale	Parents/careers reported that their child had made a friend from attending the LEGO club	8 weeks	III	Low	NC
2014	Tuonen et al.	4 (2 M; 2 F)	8–13	School	1 h/week	Video-oservation	Improved on social skills	9 weeks	III	Low	NC
2014	Boyne	6 (5 M; 1 F)	6–10	School	30 m/week	Video- observation+ SCI Inventory and the Belonging Scale	Improved on social communication skills	9 weeks	III	Low	NC
2015	Barakova et al.	6 M	8–12	Clinical	30 m/week	Video observation	Improved on social initiations and decreased instances of playing alone	4 weeks	III	Low	NC
2015	MacCormack et al.	17 M	7–12	Clinical	1 h/week	Interviews	Improved on play and socialization	8 weeks	III	Low	NC
2015	Huskens et al.	3 M ASD 3 SIB (1 M; 2 F)	5–11	Clinical	30 m/week	Video observation	Improved on collaborative behaviours	5 weeks	II	Low	Critical ^††^
2015	Yalamanchili	6 (4 M; 2 F)	5–6	School	20 m/week	ASRS	Improved on social skills	4 weeks	III	Low	NC
2016	Griffiths	7 (4 M; 3 F)	7–12	School	45 m/week	GARS-2+ semi-structured interviews	Improved on social skills	6 weeks	III	Low	NC
2016	Peckett et al.	10 M (6 ASD;4 TD)	5–16	Home	1 h/week	Phenomenological analysis	Improved family relationships	6 weeks	II	Low	Critical ^††^
2018	Hu et al.	3 M ASD 13 TD (10 M; 3 F)	4–6	School	2 h/week	Observation	Improved on social interactions	28–31 sessions	II	Low	Critical ^††^
2020	Levy and Dunsmuir	6 M ASD 12 TD	11–14	School	90 m/week	SSIS	Improved social engagement and frequency of social initiations	12 sessions	II	Low	Critical ^††^

^1^ Johns Hopkins Nursing Evidence-Based Practice (JHNEBP) rating scale [39] (Low and Good refer to quality of evidence). ^2^ RoB-2 ^†^ [40] was used for RCT and ROBINS-I ^††^ was used for No RCTs [41] (Low and Critical refer to risk of bias). Legend: M = Males; F = Females; ASD = Autism Spectrum Disorder; exp = experimental group; ctr = control group; h = hour; m = minutes; ASRS = Autism Spectrum Rating Scales; GARS = Gilliam Autism Rating Scale; GARS-SI = Gilliam Autism Rating Scale—Social Interaction; NC = Not Classifiable with RoB-2 or ROBINS-I; QoE = Quality of Evidence; RCT = Randomized Controlled Trials; RoB = Risk of Bias; RoB-2 = Risk of Bias-2; ROBINS = Risk of Bias In Non-Randomized studies; SCI = Social Competence Inventory; SIB = Siblings; SoE = Strength of the Evidence; SSIS = Social Skills Improvement System; TD = Typical Developing; VABS = Vineland Adaptive Behavior Scale; VABS-SD = Vineland Adaptive Behavior Scale—Social Development.

## Supplementary Materials

The following are available online at https://www.mdpi.com/article/10.3390/brainsci11060702/s1, Table S1: PRISMA 2009 CHECKLIST, Table S2: Search Strategies, Table S3: JHNEBP classification, Table S4: RoB-2—Risk of Bias for the RCT included in the systematic review, Table S5: ROBINS-I—Risk of Bias for the no-RCTs included in the systematic review.
