# Supplementary material for: Could You Give Me the Blue Brick? LEGO®-Based Therapy as a Social Development Program for Children with Autism Spectrum Disorder: A Systematic Review
Source: Brain Sci. 2021 May 26;11(6):702. doi: 10.3390/brainsci11060702 (PMC8228619; doi:10.3390/brainsci11060702)
Supplement: Supplementary file 1 [file brainsci-11-00702-s001.zip › brainsci-1197962-supplementary.pdf]

brain sciences

MDPI

# TABLE S1 – PRISMA 2009 CHECKLIST

| Section/topic | # | Checklist item | Reported on page # |
|---|---|---|---|
| **TITLE** | | | |
| Title | 1 | Could you give me the blue brick? The LEGO®-based therapy as social development program for children with Autism Spectrum Disorder: a systematic review | 1 |
| **ABSTRACT** | | | |
| Structured summary | 2 | LEGO®-based therapy is a social skills development program aimed to children with autism spectrum disorder (ASD). A systematic review of the literature was conducted using PRISMA guidelines. PubMed, Scopus and Web of Science bibliographic databases were searched from their date of inception to August 2020. The review included 19 articles. Studies were classified according to experimental designs (e.g. Randomized Control Trial, waiting least list control group; case-control) and a narrative synthesis of each was provided, along with a critical discussion on strengths and weaknesses of the available literature on the topic. The findings of LEGO®-based therapy studies should be interpreted and generalized with caution, in light of the several limitations we identified as well as the presence of only one relative paucity of randomized controlled trials and the small sample sizes of included studies. | 1 |
| **INTRODUCTION** | | | |
| Rationale | 3 | The first documented experience of LEGO®-based therapy was described by two studies published in 1995. Altman and Esber first used LEGO®-based therapy on nine adolescents (11-16 years old) with severe disruptive behavior disorders: in this seminal work, the authors underlined the effects of this intervention on focus attention and collaborative skills. Kohler and colleagues adopted manipulative play activities, including LEGO bricks, with 3 preschoolers with ASD and 6 of their typical classmates, in order to study the group-oriented contingency to increase social interactions between children with ASD and their peers. This study showed that the interdependent group contingency improved the socio-interactive exchanges between children with ASD and peers. Since then, a discrete | 2 |

| | | | |
|---|---|---|---|
| | | amount of LEGO®-based therapy studies has been conducted; however, evidence on the efficacy of this kind of intervention is still poorly understood and has not been summarized yet. | |
| Objectives | 4 | The main aim of our review is to summarize the available data of the LEGO®-based therapy studies to understand the effect on social skills in children with ASD. Studies aimed at assessing the effects of LEGO®-based therapy on children with ASD by means of pre- to post-treatment comparisons and providing quantitative outcomes, based on clinical measures of social skills were included. | 3 |
| **METHODS** | | | |
| Protocol and registration | 5 | The protocol of our systematic review was not preregistered on an international database such as PROSPERO. | – |
| Eligibility criteria | 6 | Exclusion criteria were as follows: (1) no quantitative outcome measures were provided; ; (i.e. studies providing qualitative results based on clinical observations); (2) articles published in a language other than English; (3) studies providing protocol designs ra-ther than clinical interventions; (4) reviews. | 3 |
| Information sources | 7 | PubMed, Scopus and Google Scholar bibliographic databases were searched from their date of inception to August 2020. Reference lists of all included studies were also searched for further relevant citations. | 3 |
| Search | 8 | The authors discussed and reviewed the results of an initial scoping search and finally used the following search strategy: (LEGO therapy) AND (autism OR autistic AND disorder); the full search strategy is available in Supplementary Table 2. | 3 |
| Study selection | 9 | The search included reviews and original studies and, if a previous review was found, the reference list was searched to identify and retrieve the primary studies. Abstracts were retrieved using our search strategy and duplicates were then removed. If a title appeared potentially eligible but no abstract was available, the full-text article was retrieved. Three researchers (AN,RM and and GS) scanned all titles and abstracts to identify relevant articles for full-text retrieval. Any disagreements were resolved by consensus. | 3 |
| Data collection process | 10 | Three researchers (AN, RM and GS) independently extracted relevant data from full-text articles. Any disagreements were resolved by consensus. When datasets of any study were not fully available, authors were contacted to attain the required data and include all possible studies. | 3 |

| | | | |
|---|---|---|---|
| Data items | 11 | Items extracted from full-text articles included year of publication, authors, sample size, age of participants, intervention setting, treatment duration and number of sessions, assessment tools and outcome results. Outcome variables included the Gilliam Autism Rating Scale – social interaction (GARS–SI) subscale score, the Autism Spectrum Rating Scales – social/communication (ASRS – SC) subscale score, the frequency of Self-Initiated Social Contacts (SISC) and the Duration of Social Interactions (DSI). | 3 |
| Risk of bias in individual studies | 12 | The risk of bias in individual studies and the quality of each article were ascertained using the Johns Hopkins Nursing Evidence-Based Practice (JHNEBP) rating scale. | 3 |

| | | | |
|---|---|---|---|
| **RESULTS** | | | |
| Study selection | 13 | Identification and selection of papers started from 554 abstracts that were retrieved using our search strategy. First, 16 abstracts were removed as duplicates, thus 39 were carefully screened. Twenty records were excluded based on abstract or title. The remaining 19 full-text studies were included in the narrative review and subdivided according to the type of study and level of evidence | 4 |
| Study characteristics | 14 | For each included study, sample sizes, demographic data (age and gender) and full-scale intelligence quotient (FSIQ) were extracted from full-text papers, along with details on the intervention (duration in months, social interaction outcome variables). | 4 |
| Risk of bias within studies | 15 | Risk of bias within studies, as ascertained using the Johns Hopkins Nursing Evidence-Based Practice (JHNEBP) rating scale, ROBIN-I and RoB-2 is reported in Table 1 | 4 |
| Synthesis of results | 16 | A narrative synthesis of the studies is reported in the Results section | 11 |
| **DISCUSSION** | | | |
| Summary of evidence | 17 | Our systematic review highlighted that the evidence on the effectiveness of LEGO®-based therapy is still limited. However, the available studies reported an improvement in social skills following an intervention with LEGO®-based therapy, especially in terms of frequency of self-initiated contacts and duration of social interactions, as measured by commonly used scales of socio-interactional skills (i.e. GARS and ASRS). | 12 |
| Limitations | 18 | The findings of LEGO®-based therapy studies should be interpreted in light of several limitations: (1) only one few randomized controlled studies; (2) different measures of social skills used in the different studies; (3) small sample | 13 |

| | | | |
|---|---|---|---|
| | | size of the studies; (4) variability in terms of clinical and socio-demographic characteristics; (5) relatively narrow search strategy; (6) the systematic review has been focused only on outcomes related to social interaction, but other outcomes that might benefit from LEGO therapy could be potentially evalu-ated (e.g. joint attention, fine motricity, etc.). (7) the protocol of our systematic review was not preregistered. | |
| Conclusions | 19 | The usability of LEGO®-based therapy in different settings can be considered the strength of this approach and the possibility of involving also typically developing peers and parents may represent a naturalistic learning and sharing opportunity for people with ASD. However, more clinical studies need to be conducted to make the LEGO®-based therapy as a recommended evidence-based intervention. | 13 |
| **FUNDING** | | | |
| Funding | 20 | This research was funded by the Italian Ministry of the Health RC2019 and 5 × 1000 funds. | 13 |

**Table S2 - Search Strategies**

| | ***Search Lines*** | ***Fields*** | ***Restrictions*** |
|---|---|---|---|
| **PubMed** | (LEGO therapy) AND (autism OR autistic AND disorder) | Title OR Abstract | None |
| **Scopus** | (LEGO therapy) AND (autism OR autistic AND disorder) | Title, abstracts, keywords | None |
| **Web of Science** | (LEGO therapy) AND (autism OR autistic AND disorder) | All | None |

| Year | Author | n°Reference | Study design | Classification of the study design according to JHNEBP[1] | SoE | QoE |
|---|---|---|---|---|---|---|
| 2004 | LeGoff | 23 | repeated-measures, waiting list control design | Quasi-experimental study | Level II | Low |
| 2006 | LeGoff & Sherman | 28 | Non-Randomized Studies of Interventions | Quasi-experimental study | Level II | Good |
| 2008 | Owens et al. | 29 | RCT | Experimental study/(RCT) | Level I | Good |
| 2010 | Pang | 52 | Case report (n=1) | Case studies | Level V | Low |
| 2010 | Wainer et al. | 53 | Cases series (n=7) | Non-experimental study | Level III | Low |
| 2012 | Andras | 46 | waiting list control design | Quasi-experimental study | Level II | Low |
| 2012 | Andrews et al. | 54 | Case report (n=1) | Case studies | Level V | Low |
| 2013 | Brett | 47 | within-subjects baseline design | Quasi-experimental study | Level II | Low |
| 2014 | Evans et al. | 55 | Case series (n=18) | Non-experimental study | Level III | Low |
| 2014 | Tuonen et al. | 56 | Cases series (n=4) | Non-experimental study | Level III | Low |
| 2014 | Boyne | 57 | Cases series (n=6) | Non-experimental study | Level III | Low |
| 2015 | Barakova et al. | 58 | Case series (n=6) | Non-experimental study | Level III | Low |
| 2015 | MacCormack et al. | 59 | Case series (n=17) | Non-experimental study | Level III | Low |
| 2015 | Huskens et al. | 48 | Non-Randomized Studies of Interventions | Quasi-experimental study | Level II | Low |
| 2015 | Yalamanchili | 60 | Case series (n=6) | Non-experimental study | Level III | Low |
| 2016 | Griffiths | 61 | Case series (n=7) | Non-experimental study | Level III | Low |
| 2016 | Peckett et al. | 49 | Non-Randomized Studies of Interventions | Quasi-experimental study | Level II | Low |
| 2018 | Hu et al. | 50 | Non-Randomized Studies of Interventions | Quasi-experimental study | Level II | Low |
| 2020 | Levy and Dunsmuir | 51 | Non-Randomized Studies of Interventions | Quasi-experimental study | Level II | Low |

**Table S3 – JHNEBP classification**

**[1]RCT=Groups were randomly assigned; Quasi-experimental=Groups were not randomly assigned; Non-experimental=there is no control group; Case studies=studies with a single subject**

*Ref: Newhouse, R., Dearholt, S., Poe, S., Pugh, L. C., White, K. M. Evidence-based practice: A practical approach to implementation. Journal of Nursing Administration, 2005, 35 (1), 35–40.*

**Table S4 - RoB-2 - Risk of Bias for the RCT included in the systematic review**

| Year | Author | Random sequence | Effect of assignment to intervention | Effect of adhering to intervention | Missing outcome data | Measurement of the outcome | Selection of the reported result |
|---|---|---|---|---|---|---|---|
| 2008 | Owens et al. | Low | Low | Low | Low | Low | Low |

*Ref: Sterne, J.A.C., Savović, J., Page, M.J., Elbers, R.G., Blencowe, N.S., Boutron, I., Cates, C.J., Cheng, H,Y., Corbett, M.S., Eldridge, S.M., Hernán, M.A., Hopewell, S., Hróbjartsson, A., Junqueira, D.R., Jüni, P., Kirkham, J.J., Lasserson, T., Li, T., McAleenan, A., Reeves, B.C., Shepperd, S., Shrier, I., Stewart, L.A., Tilling, K., White, I.R., Whiting, P.F., Higgins, J.P.T. RoB 2: a revised tool for assessing risk of bias in randomised trials. BMJ, 2019, 366, l4898.*

**Table S5 - ROBINS-I - Risk of Bias for the no-RCTs included in the systematic review**

| Year | Author | Confounding | Selection of partecipants | Classification of interventions | Deviations from intended interventions | Missing data | Measurement of outcomes | Selection of reported results | Overall |
|---|---|---|---|---|---|---|---|---|---|
| 2006 | LeGoff & Sherman | Low | Low | Low | Moderate | Low | Low | Low | Moderate |
| | | | | | | | | | |
| 2015 | Huskens et al. | Critical | Critical | Critical | Critical | Critical | Critical | Serious | Critical |
| | | | | | | | | | |
| 2016 | Peckett et al. | Serious | Serious | Critical | Critical | Critical | Critical | Critical | Critical |
| | | | | | | | | | |
| 2018 | Hu et al. | Serious | Serious | Critical | Critical | Critical | Critical | Critical | Critical |
| | | | | | | | | | |
| 2020 | Levy and Dunsmuir | Serious | Serious | Critical | Critical | Critical | Critical | Critical | Critical |

*Ref: Sterne, J.A.C., Hernán, M.A., Reeves, B.C., Savović, J., Berkman, N.D., Viswanathan, M., Henry, D., Altman, D.G., Ansari, M.T., Boutron, I., Carpenter, J.R., Chan, A.W., Churchill, R., Deeks, J.J., Hróbjartsson, A., Kirkham, J., Jüni, P., Loke, Y.K., Pigott, T.D., Ramsay, C.R., Regidor, D., Rothstein, H.R., Sandhu, L., Santaguida, P.L., Schünemann, H.J., Shea, B., Shrier, I., Tugwell, P., Turner, L., Valentine, J.C., Waddington, H., Waters, E., Wells, G.A., Whiting, P.F., Higgins, J.P.T. ROBINS-I: a tool for assessing risk of bias in non-randomized studies of interventions. BMJ, 2016, 355, i4919.*
